# CaliDent-Net: domain-constrained self-supervised pre-training with parallel attention and prototype calibration for dental radiograph analysis

**DOI:** 10.3389/fmed.2026.1881041

**Published:** 2026-09-01

**Authors:** Yanyu Miao, Xiaoqin Zhang, Fei Leng, Yanling Zhu

**Affiliations:** 1 School of Stomatology, Jiangxi Medical College, Nanchang University, Nanchang, China; 2 Jiangxi Province Key Laboratory of Oral Biomedicine, Nanchang, China; 3 Jiangxi Province Clinical Research Center for Oral Diseases, Nanchang, China; 4 The Second Affiliated Hospital of Jiaxing University, Jiaxing, China

**Keywords:** artificial intelligence in dentistry, deep learning, dental radiograph analysis, diagnostic decision support, explainable AI, self-supervised learning

## Abstract

Automated interpretation of dental radiographs is limited by the shortage of expert-annotated training images and by standard softmax classifiers that often assign confidence values that do not reflect empirical accuracy. We introduce *CaliDent-Net*, a unified framework that integrates and domain-adapts three established techniques contrastive self-supervision, dual attention, and prototype-based classification to obtain four clinically relevant properties in a single pipeline: data efficiency, probabilistic calibration, case-based interpretability, and CPU-level inference. CaliDent-Net consists of a Radiograph-Aware Contrastive Encoder (RACE), which performs pre-training with augmentations that respect tooth and bone anatomy; a Parallel Recalibration Attention Block (PRAB), which computes spatial saliency and channel importance through two forward-independent pathways before learned fusion; and a Similarity-Scored Prototype Classifier (SSPC), which replaces the unconstrained linear head with bounded cosine-prototype scoring. SSPC mitigates logit inflation, supports nearest-prototype retrieval as a case-based explanation mechanism, and improves calibration at the architectural level rather than relying only on *post-hoc* correction. We evaluate CaliDent-Net on the public Dental Radiography benchmark (1,272 images, four pathological classes) against eight deep learning baselines under matched protocols. The proposed model achieves 96.4% accuracy and a macro-averaged AUC of 0.984, representing an improvement of 2.3 to 9.5 percentage points over the baselines. Its Brier score (0.021) and expected calibration error (0.013) are 45% and 71% lower than the ResNet-50 reference (0.038 and 0.046), with only a minor portion of the calibration gain attributable to temperature scaling. With 40% of the labels (about 356 images), CaliDent-Net exceeds the full-data ResNet-50 baseline, and its CPU inference time is 214 ms per image at 2.8 GFLOPs. The findings support an integrative recipe rather than a new learning primitive, but they do not establish clinical translation; multi-site prospective validation, blinded multi-rater interpretability evaluation, and domain-shift robustness assessment remain necessary.

## 1 Introduction

Oral disease constitutes one of the most pervasive non-communicable health burdens worldwide, affecting an estimated 3.5 billion individuals across every socioeconomic stratum and geographic region (1–3). Untreated dental caries alone compromises the quality of life of approximately 2.3 billion people, and its downstream sequelae including periodontal disease, periapical infection, and eventual tooth loss have been epidemiologically linked to systemic conditions such as cardiovascular disease and adverse obstetric outcomes (2). Early and accurate radiographic diagnosis is therefore the principal mechanism by which preventable pathology is intercepted before it progresses to irreversible structural damage.

Dental radiography (periapical, bitewing, and panoramic projections) remains the primary biomedical imaging modality for identifying carious lesions, assessing restoration integrity, detecting impacted teeth, and evaluating alveolar bone support (4). Accurate interpretation requires extended specialist training, and inter-observer agreement is known to be variable, particularly for mixed-pathology cases and incipient lesions. In primary-care and resource-limited settings where specialist expertise is scarce, delayed or inaccurate diagnoses translate directly into avoidable morbidity and more costly downstream treatment.

The application of artificial intelligence (AI) and computational image analysis to dental radiograph interpretation has expanded steadily over the past decade, with convolutional neural network (CNN) pipelines achieving benchmark accuracies competitive with expert performance on targeted tasks (5–7). Two structural limitations nonetheless continue to constrain clinical translation. First, dominant deep learning architectures require large annotated corpora, typically thousands of meticulously labeled images, a requirement that most clinical institutions cannot satisfy given the high per-image annotation cost and the inherent scarcity of rare pathological categories (8). Second, conventional softmax classifiers tend to yield systematically overconfident posterior probability estimates that fail to reflect true empirical uncertainty (9). A computational diagnostic assistant that assigns 93% confidence to predictions that are correct only 68% of the time does not support clinical reasoning; rather, it actively erodes patient safety by communicating false certainty.

Recent advances in self-supervised contrastive representation learning, spatially aware attention mechanisms, and prototype-based classification offer principled strategies for jointly addressing both limitations (10–13). Each approach has received individual attention within medical image informatics, but, to the best of our knowledge, their integrated deployment within a unified, data-efficient dental AI framework has not yet been demonstrated; Table 1 systematically documents this gap.

**Table 1:** Positioning of CaliDent-Net against representative prior work.

Study	Year	Dataset/modality	Method	*N*_train_	SSL	Calib.	Interp.
Lee et al. (7)	2018	Periapical	CNN + transfer learning	3,000	×	×	×
Yüksel et al. (5)	2021	Panoramic	DENTECT (detection + enum.)	1,005	×	×	×
Baydar et al. (6)	2024	Bitewing	U-Net + classification	609	×	×	Partial^†^
Alsakar et al. (14)	2024	Dental Radiography	MobileNetV2 + Swin + bagging	1,272	×	×	×
Elazab et al. (15)	2026	Dental radiography	DeiT + CoAtNet + stacking	1,272	×	×	×
CaliDent-Net (ours)	2026	Dental radiography	RACE + PRAB + SSPC	1,272	✓	✓	✓

SSL, self-supervised learning; Calib. calibration metrics reported; Interp., = interpretability mechanism provided; *N*_train_ is the labeled training-set size. ✓ = addressed; × = not addressed. ^†^ “Partial” interpretability: lesion-level segmentation masks are produced by the U-Net stage, but no global or class-level explanation mechanism is offered.

The objective of this study is to demonstrate that data efficiency, probabilistic calibration, case-based interpretability, and computational accessibility are not mutually exclusive design objectives and that careful architectural integration can deliver all four concurrently. The proposed framework is developed and evaluated on the publicly available Dental Radiography benchmark (14) (1,272 images, four classes) and is positioned as a decision-support tool for clinicians rather than a substitute for clinical judgement. Translation to routine clinical deployment remains subject to prospective validation and to regulatory pathways (e.g., FDA Software as a Medical Device or CE marking under the EU MDR/AI Act) that fall outside the scope of the present study.

The key contributions of this work are as follows:

- A Radiograph-Aware Contrastive Encoder (RACE) that pretrains the backbone with an augmentation set restricted to operations consistent with dental anatomy. Vertical flips, large-angle rotations, and strong color or saturation jitter are excluded because they violate the fixed superior–inferior orientation of dentition and disturb the grayscale contrast cues on which diagnostic reading depends. The contrastive objective itself is conventional (InfoNCE under a SimCLR-style formulation); what we examine is the effect of narrowing the invariance set to transformations that respect these two structural priors.
- A Parallel Recalibration Attention Block (PRAB) in which channel importance and spatial saliency are produced by two branches that, on the forward pass, are computed independently from a common input and then combined through a learned 1 × 1 projection. The motivation is structural: In CBAM, the two branches are applied in sequence, so the spatial branch never sees the original backbone activations, only their channel-reweighted version. PRAB instead feeds both streams from the same feature map. The independence applies to the forward computation; on the backward pass, gradients from the shared fusion layer still couple the two branches.
- A Similarity-Scored Prototype Classifier (SSPC) that substitutes cosine-similarity scoring against learnable class prototypes for the usual unconstrained linear head. Because similarities are confined to [−1, 1], logit growth is suppressed, calibration is improved at the architectural level (not only through *post-hoc* rescaling), and predictions admit a case-based reading via nearest-prototype retrieval.
- A composite training objective that pairs cross-entropy with two regularisers acting on the prototypes, one for inter-prototype separation and one for intra-class compactness. These ingredients are not new in isolation; their function here is to shape the embedding geometry on which the SSPC depends.

Each of the three modules builds on mature research trajectories: contrastive self-supervision, channel and spatial attention, and prototype-based classification. None is offered here as a new learning primitive. Our core contribution lies in their integration. Combining a domain-constrained encoder, a forward-independent attention block, and a bounded-logit prototype head into one pipeline yields data efficiency, calibration, interpretability, and CPU deployability within the same model, and we examine how the three components interact on a dental-radiograph benchmark. To our knowledge, no earlier work on dental radiographs has jointly reported calibration metrics (ECE and Brier score), used domain-specific self-supervised pretraining, and employed a prototype-based head within a single framework.

The remainder of the study is organized as follows. Section 2 reviews the related literature. Section 3 describes the methodology. Section 4 describes the dataset, preprocessing, and ethics and also presents the experimental protocol. Section 5 reports results. Section 6 discusses implications, limitations, and failure modes. Section 7 concludes.

## 2 Related work

### 2.1 Deep learning for dental radiograph diagnosis

CNN-based dental image analysis has matured steadily over the past decade. Lee et al. (7) showed that CNNs trained on periapical images can reach caries detection sensitivities above 82%. Baydar et al. (6) coupled U-Net segmentation with classification on bitewing radiographs, providing a lesion-localization map at the cost of additional annotation effort. Yüksel et al. (5) proposed DENTECT, a multi-stage pipeline that detects dental treatments and performs tooth enumeration on panoramic images. Alsakar et al. (14) fused MobileNetV2 and Swin Transformer features with bagging, reaching 95.6% accuracy on multi-label dental classification; we adopt their benchmark and compare directly with that result. Elazab et al. (15) combined DeiT and CoAtNet through a cross-attention mechanism and a stacking classifier. That paper was published concurrently with this work, and the comparison below uses the results as reported in the published version. Ensemble pipelines of this kind raise inference cost considerably and limit CPU deployment. We conducted a targeted search of PubMed, IEEE Xplore, and Scopus using the terms (dental OR radiograph) and (ECE OR Brier OR calibration) and found no prior dental radiograph study that jointly reports ECE/Brier, uses domain-specific self-supervised pre-training, and employs prototype-based classification. CaliDent-Net closes all three gaps within a single framework.

### 2.2 Attention mechanisms in medical image analysis

The Squeeze-and-Excitation (SE) network (16) introduced learned channel-wise recalibration by pairing global pooling with a compact gating function. CBAM (12) extended this to joint channel-spatial attention but applied the two branches sequentially: Channel recalibration runs first, and then, spatial recalibration operates on the already-reweighted feature map. On the forward pass, this ordering means that the spatial branch never sees the original backbone features; it sees only their channel-reweighted version. This is a direct architectural observation rather than a claim about gradient independence, and it motivates PRAB (Section 3.4), which routes both streams from the same input map. Post-CBAM work on dual attention (for example DANet and variants of self-attention for dense prediction) has largely targeted segmentation tasks; survey evidence continues to support the view that attention-guided designs outperform non-attention counterparts for small and localized pathologies, a finding that applies directly to early carious lesions and subtle periapical changes (17).

### 2.3 Self-supervised and data-efficient learning

SimCLR (10) established that contrastive InfoNCE pretraining transfers reliably to a wide range of downstream tasks. MoCo (11) introduced momentum contrast for stable training with smaller batch sizes, and BYOL (18) showed that useful representations can be learned without explicit negative pairs. Mask-based methods such as MAE (19) and self-distillation methods such as DINO (20) have since become influential in medical imaging. We retained an InfoNCE/SimCLR formulation for RACE because the contrastive family has the most direct mechanism for incorporating domain-constrained augmentations (the pretext task is the invariance to an explicit set of transformations), which is the central design idea of this paper. Raghu et al. (21) found that self-supervised pre-training frequently matches or exceeds ImageNet-supervised initialization when labeled samples are scarce, which is consistent with the data-efficiency regime targeted here.

### 2.4 Prototype learning, calibration, and alternatives

Generalized Learning Vector Quantization (22) set out the principle of learning class-representative vectors under margin-based objectives. ProtoPNet (13) extended the idea to modern deep networks and showed that prototype-grounded predictions support more meaningful human-AI interaction in image classification; subsequent work (ProtoPool, Deformable ProtoPNet) has generalized the prototype head to pools and deformable parts and is discussed in Section 6 as a future direction. Guo et al. (9) demonstrated that modern networks are systematically overconfident and that *post-hoc* temperature scaling corrects miscalibration without addressing its architectural source. Label smoothing and focal loss are architectural-level alternatives that have been applied to calibration: Both shape the target distribution or the loss landscape rather than the logit range. CaliDent-Net addresses the problem further upstream, by bounding the logit space itself through cosine normalization, and uses temperature scaling as a final refinement.

### 2.5 Positioning, external validity, and interpretability context

The proposed model adapts techniques that are already well established, and the originality of the present study lies instead in the way these techniques are combined for a single clinical task. As far as we are aware, accuracy, calibration, interpretability, and computational cost have not previously been reported in combination for dental radiographs (Table 1); it is this joint reporting, rather than any individual component, that we regard as our contribution.

Every result reported below comes from a single public dataset of 1, 272 images, of which 191 form the test split. Because these figures were obtained only after self-supervised pretraining, hyperparameter tuning, and oversampling of the minority class, they may reflect optimization toward this particular dataset rather than genuine clinical performance; the tendency of medical-imaging models to deteriorate once the scanner, exposure, or patient population changes is by now well documented (23). Therefore read our findings as a demonstration of technical viability and not as evidence of clinical generalization, and we defer a fuller account of the safeguards adopted against overfitting, together with their limitations, to Section 6.

The interpretability of the model can be approached from two directions, which are complementary rather than competing. The prototype head supplies what is commonly termed a case-based explanation: It reports that a given radiograph resembles particular labeled exemplars, an idea that goes back to ProtoPNet (13). The second and more familiar approach instead produces pixel-attribution maps. Grad-CAM (24) propagates class gradients backward to mark the regions that most influenced a prediction, whereas LIME (25) and SHAP (26) fit local surrogate models toward the same end. Because the two families answer genuinely different questions (which earlier cases justify a decision, as opposed to which pixels produced it), we report both rather than relying on prototype retrieval alone.

## 3 Methodology

### 3.1 Problem formulation

**Figure 1:**
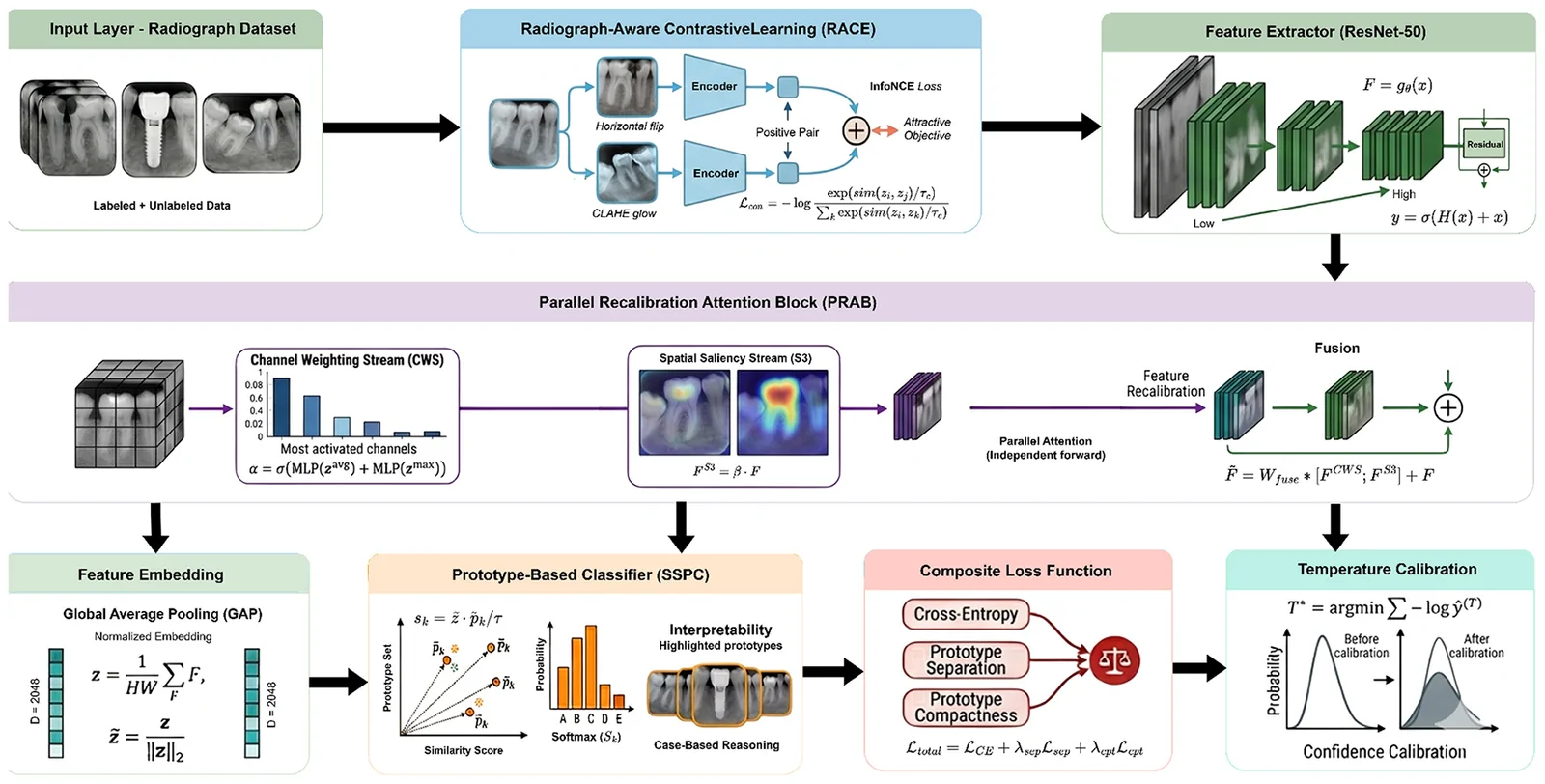
Schematic of the CaliDent-Net framework.

The overall architecture of the proposed framework is illustrated in Figure 1. Let $D_{L}={\left\{\left(x_{i},y_{i}\right)\right\}}_{i=1}^{N}$ denotes a labeled dataset of *N* dental radiographs, where $x_{i}\in {\mathbb{R}}^{C\times H\times W}$ is an input image and *y*_*i*_∈{1, …, *K*} is one of *K* = 4 diagnostic class labels (cavities, fillings, implants, impacted teeth). An unlabeled set $U={\left\{u_{j}\right\}}_{j=1}^{M}$ is drawn strictly from the training partition with labels withheld, so that no validation or test data leak into pretraining; in this study, *M* equals the size of the training partition (Section 4.1). The end-to-end forward pass is


$$\begin{array}{c} ŷ=\text{SSPC}\left(\text{GAP}\left(\text{PRAB}\left(g_{\theta }\left(x\right)\right)\right)\right), \end{array}$$ (1)

where *g*_θ_(·) is the backbone encoder, and SSPC is the cosine prototype head of Section 3.6. The full training procedure is given in Algorithm 1.

Algorithm 1 CaliDent-Net. 
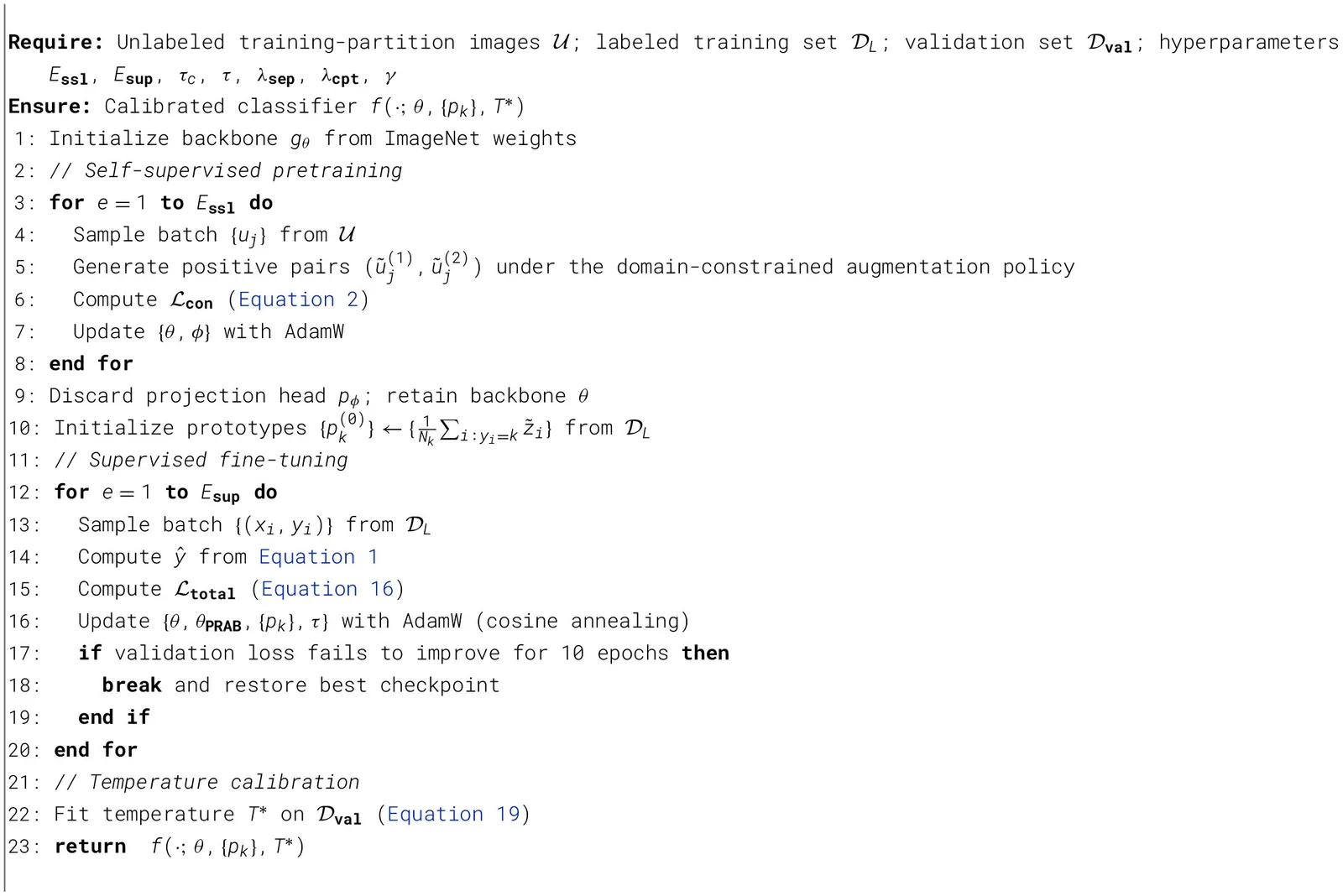

### 3.2 Radiograph-Aware Contrastive Encoder (RACE)

Dental radiographs have two structural properties that standard contrastive augmentation policies routinely violate. First, dental anatomy has a fixed superior-inferior orientation: Root apices point away from the occlusal plane, so vertical flips or rotations beyond 15° produce anatomically impossible views. Second, diagnostically critical information is carried by high-contrast grayscale relationships between enamel, dentine, cementum, and alveolar bone, and aggressive color or saturation jitter corrupts those relationships. These exclusions were reviewed and endorsed by a clinical with dental imaging experience prior to implementation.

For each unlabeled image *u*_*j*_, two independent branches produce a positive pair $\left({ũ}_{j}^{\left(1\right)},{ũ}_{j}^{\left(2\right)}\right)$. Permitted augmentations are random cropping [scale [0.8, 1.0], aspect ratio [0.75, 1.33]], and horizontal flipping (*p* = 0.5). Excluded are vertical flipping, rotations beyond 15°, saturation jitter, hue jitter, and random grayscale. The policy extends the principle of domain-informed augmentation selection (27) to the orientation and contrast constraints of dental radiographs. All other preprocessing, including CLAHE, is applied once as a deterministic preprocessing step before training (Section 4.1); no stochastic CLAHE is applied in the augmentation pipeline, so the contrast of a given radiograph is fixed across the two positive views.

A shared backbone *g*_θ_(·) maps augmented views to *h*∈ℝ^*d*^, which a two-layer projection head *p*_ϕ_ maps to $z\in {\mathbb{R}}^{d_{z}}$ (*d*_*z*_ = 256, with batch normalization after the hidden layer). The InfoNCE objective (10) is


$$\begin{array}{c} L_{\text{con}}=-\frac{1}{B}\overset{B}{\underset{j=1}{\sum }}\log\frac{\exp\left(\mathrm{sim}\left(z_{j}^{\left(1\right)},z_{j}^{\left(2\right)}\right)/\tau_{c}\right)}{\sum_{k=1}^{B}\exp\left(\mathrm{sim}\left(z_{j}^{\left(1\right)},z_{k}^{\left(2\right)}\right)/\tau_{c}\right)}, \end{array}$$ (2)

where $\mathrm{sim}\left(a,b\right)=a^{⊤}b/\left(||a|{|}_{2}||b|{|}_{2}\right)$ and τ_*c*_ is the contrastive temperature. After pretraining, *p*_ϕ_ is discarded and θ is transferred to the supervised stage.

### 3.3 Backbone architecture

The encoder *g*_θ_ is a standard ResNet-50 (28). We retain ResNet-50 over lighter alternatives (MobileNetV3, EfficientNet-Lite) because its bottleneck structure integrates cleanly with the PRAB insertion point between stages 3 and 4 and because its 2,048-dimensional embedding space is a familiar target for prototype-based heads. The lighter alternatives are reported as baselines in Section 4. Following He et al. (28), each of the four stages ϕ_1_, …, ϕ_4_ is a stack of bottleneck blocks (*n*_1_, *n*_2_, *n*_3_, *n*_4_ = 3, 4, 6, 3). We insert the PRAB between ϕ_3_ and ϕ_4_, operating on the richest intermediate representation before final semantic abstraction; alternative placements (after ϕ_4_, or dual placement) were evaluated during development and produced smaller accuracy gains at comparable compute cost.

### 3.4 Parallel Recalibration Attention Block (PRAB)

The PRAB receives the stage-3 feature map *F*∈ℝ^*C*×*H*^′ × *W*′ and routes it through two structurally independent branches: the Channel Weighting Stream (CWS) and the Spatial Saliency Stream (S3). Both branches operate on the original *F* with no cross-stream information exchange on the forward pass. During backpropagation, gradients from the loss still flow back through the shared fusion layer *W*_fuse_ into both branches, so the two streams are not globally independent in the backward pass; what we remove is the serial forward dependency of the CBAM design, in which the spatial branch operates on an already-reweighted feature map.

#### 3.4.1 Channel weighting stream

Dual global pooling yields the max-pooled feature descriptor, as defined in Equations 3, 4, respectively


$$\begin{array}{cc} z_{c}^{\text{avg}} & =\frac{1}{H^{\prime }W^{\prime }}\sum_{i,j}F_{c,i,j}, \end{array}$$ (3)

$$\begin{array}{cc} z_{c}^{\text{max}} & =\max_{i,j}F_{c,i,j}. \end{array}$$ (4)

Both *z*^**avg**^*andz*^**max**^∈ℝ^*C*^ pass through a shared bottleneck Multi-Layer Perceptron with a reduction ratio *r*, which is formulated in Equation 5:


$$\begin{array}{c} \text{MLP}\left(z\right)=W_{2}^{\text{ch}}\cdot \delta \left(W_{1}^{\text{ch}}\cdot z\right), \end{array}$$ (5)

where $W_{1}^{\text{ch}}\in {\mathbb{R}}^{C/r\times C}$, $W_{2}^{\text{ch}}\in {\mathbb{R}}^{C\times C/r}$, and δ(·) is ReLU. The channel attention weight is to compute the final channel attention weight, as calculated in Equation 6


$$\begin{array}{c} \alpha =\sigma \left(\text{MLP}\left(z^{\text{avg}}\right)+\text{MLP}\left(z^{\text{max}}\right)\right)\in {\mathbb{R}}^{C}, \end{array}$$ (6)

and the recalibrated map is $F_{c,i,j}^{\text{CWS}}=\alpha_{c}\cdot F_{c,i,j}$.

#### 3.4.2 Spatial saliency stream

Channel-aggregated spatial descriptors are computed from the original *F* independently of the CWS, as defined in Equations 7, 8:


$$\begin{array}{cc} M_{i,j}^{\text{avg}} & =\frac{1}{C}\sum_{c}F_{c,i,j}, \end{array}$$ (7)

$$\begin{array}{cc} M_{i,j}^{\text{max}} & =\max_{c}F_{c,i,j}. \end{array}$$ (8)

After concatenation *M* = [*M*^avg^; *M*^max^]∈ℝ^2 × *H*^′ × *W*′, the spatial attention weight is computed using a 7 × 7 convolutional layer, as formulated in Equation 9


$$\begin{array}{c} \beta =\sigma \left(\text{Con}{\text{v}}_{7\times 7}\left(M\right)\right)\in {\mathbb{R}}^{1\times H^{\prime }\times W^{\prime }}. \end{array}$$ (9)

The recalibrated map is $F_{c,i,j}^{\text{S}3}=\beta_{i,j}\cdot F_{c,i,j}$.

#### 3.4.3 Parallel fusion

Outputs are concatenated and fused through a learned projection with a residual skip connection, as defined in Equation 10:


$$\begin{array}{c} \tilde{F}=W_{\text{fuse}}*\left[F^{\text{CWS}};F^{\text{S}3}\right]+F, \end{array}$$ (10)

where $W_{\text{fuse}}\in {\mathbb{R}}^{C\times 2C\times 1\times 1}$ restores the *C*-channel representation. The additional parameter count introduced by PRAB is approximately 2*C*^2^/*r*+2·7^2^+2*C*^2^ per insertion point, which, at *C* = 1, 024 and *r* = 16 (PRAB between stages 3 and 4), is approximately 2.2 M parameters; this is comparable to a CBAM block at the same position (approximately 2.1 M) and slightly larger than a plain SE block (approximately 0.13 M).

### 3.5 Feature embedding

Global average pooling is applied after stage 4, as formulated in Equation 11:


$$\begin{array}{c} z=\frac{1}{H_{4}W_{4}}\underset{i,j}{\sum }{\tilde{F}}_{:,i,j}^{\left(4\right)}\in {\mathbb{R}}^{D},\;D=2048. \end{array}$$ (11)

Dropout (*p*_*d*_ = 0.25) is applied during training, followed by ℓ_2_-normalization, which is defined in Equation 12:


$$\begin{array}{c} \tilde{z}=z/||z{||}_{2}. \end{array}$$ (12)

### 3.6 Similarity-Scored Prototype Classifier (SSPC)

The SSPC maintains *K* learnable prototype vectors ${\left\{p_{k}\right\}}_{k=1}^{K}$ with $p_{k}\in {\mathbb{R}}^{D}$, initialized from class-conditional embedding means computed using only training-split images after RACE pre-training: $p_{k}^{\left(0\right)}=\frac{1}{N_{k}}\underset{i:y_{i}=k}{\sum }{\tilde{z}}_{i}$. At each forward pass, prototypes are ℓ_2_-normalized according to Equation 13:


$$\begin{array}{c} {\tilde{p}}_{k}=p_{k}/||p_{k}{||}_{2}. \end{array}$$ (13)

Classification scores use a learnable scalar temperature, calculated as shown in Equation 14:


$$\begin{array}{c} s_{k}=\left(\tilde{z}\cdot {\tilde{p}}_{k}\right)/\tau , \end{array}$$ (14)

and class probabilities follow by the standard softmax


$$\begin{array}{c} {ŷ}_{k}=\exp\left(s_{k}\right)/\sum_{j=1}^{K}\exp\left(s_{j}\right). \end{array}$$ (15)

Throughout the paper, the subscript on ŷ denotes the class index, and ŷ_*y*_*i*_, *i*_ is the predicted probability assigned by the network to the true class *y*_*i*_ of training example *i*.

#### 3.6.1 Calibration argument

In a standard linear classifier, logits $s_{k}=w_{k}^{⊤}z$ are unbounded, and as weight norms grow under cross-entropy training (9), softmax outputs collapse toward one-hot distributions, producing overconfidence. In the SSPC, cosine normalization ensures $\tilde{z}\cdot {\tilde{p}}_{k}\in \left[-1,1\right]$, so pre-temperature logits are bounded by a single scalar τ rather than by the *K*×*D* unconstrained weights of a linear head. Related work on cosine-softmax classifiers in face recognition and few-shot learning (29, 30) has independently reported that bounded cosine similarities produce more stable probability estimates than unconstrained linear heads, although to our knowledge the connection to calibration metrics (ECE, Brier) has not been drawn explicitly for medical imaging. The temperature τ = 10.0 (Table 2) scales the bounded similarity to the range [−0.1, 0.1], which is smaller than the typical logit magnitudes (order of units) observed in a trained linear head; the bounding therefore remains meaningful.

**Table 2:** Hyperparameter tuning.

Hyperparameter	Value	Status
Optimizer	AdamW	Fixed
Base learning rate	1 × 10^−4^	[10^−5^, 10^−2^], log scale
Weight decay	5 × 10^−4^	Fixed
Fine-tuning batch size	32	{16, 32, 64}
Self-supervised batch size	128	Fixed
Dropout probability *p*_*d*_	0.25	[0.0, 0.5]
PRAB reduction ratio *r*	16	{4, 8, 16, 32}
SSPC temperature τ	10.0	[1, 20]
Separation margin γ	0.10	[0.05, 0.30]
Loss weight λ_sep_	0.01	[10^−3^, 10^−1^], log scale
Loss weight λ_cpt_	0.005	[10^−3^, 10^−1^], log scale
RACE pre-training epochs	100	Fixed
Supervised fine-tuning epochs	100	Fixed
Contrastive temperature τ_*c*_	0.5	Fixed

#### 3.6.2 Case-based interpretability

For a query image, the predicted class is $k^{*}=\arg\underset{k}{\max}s_{k}$. The explanation is the set of *n*_*r*_ (*n*_*r*_ = 5) nearest training images to $\tilde{z}$ in the embedding space, retrieved by cosine similarity to ${\tilde{p}}_{k^{*}}$.

### 3.7 Composite training objective

The total training loss combines three terms:


$$\begin{array}{c} L_{\text{total}}=L_{\text{CE}}+\lambda_{\text{sep}}L_{\text{sep}}+\lambda_{\text{cpt}}L_{\text{cpt}}. \end{array}$$ (16)

$L_{\text{CE}}$ is the standard categorical cross-entropy computed on the probabilities in Equation 15. Prototype separation with margin is calculated using Equation 17


$$\begin{array}{c} L_{\text{sep}}=\frac{1}{K\left(K-1\right)}\overset{K}{\underset{\begin{array}{c} j,k=1\\ j\neq k \end{array}}{\sum }}\max\left(0,{\tilde{p}}_{j}\cdot {\tilde{p}}_{k}-\gamma \right), \end{array}$$ (17)

and prototype compactness is evaluated according to Equation 18


$$\begin{array}{c} L_{\text{cpt}}=\frac{1}{N_{b}}\overset{N_{b}}{\underset{i=1}{\sum }}\left(1-{\tilde{z}}_{i}\cdot {\tilde{p}}_{y_{i}}\right). \end{array}$$ (18)

The cross-entropy term drives discriminative classification; $L_{\text{sep}}$ prevents prototype collapse toward a single region of the embedding space; and $L_{\text{cpt}}$ tightens intra-class compactness, which supports calibration by ensuring that high-confidence predictions correspond to embeddings that sit close to their class prototype. Geometrically, the margin γ = 0.10 corresponds to an angular separation of arccos(0.10)≈84° between any two prototypes, so the penalty only activates when two prototypes come closer than this angle. The weights λ_sep_ = 0.01 and λ_cpt_ = 0.005 were selected by the same Optuna search as the remaining hyperparameters (Table 2).

### 3.8 Post-training temperature calibration

After supervised fine-tuning, a scalar temperature T^*^>0 is optimized on the validation set via L-BFGS-B (31):


$$\begin{array}{c} T^{*}=\arg\underset{T>0}{\min}-\underset{i\in D_{\text{val}}}{\sum }\log{ŷ}_{y_{i},i}^{\left(T\right)}, \end{array}$$ (19)

with ${ŷ}_{k,i}^{\left(T\right)}=\exp\left(s_{k,i}/T\right)/\underset{j}{\sum }\exp\left(s_{j,i}/T\right)$. Because *T* applies monotonically to every logit, the ranking $\arg\underset{k}{\max}s_{k,i}$ is invariant under temperature scaling (9); this step therefore does not alter any class prediction and affects calibration metrics only.

## 4 Experimental setup

### 4.1 Dataset and experimental protocol

Experiments were conducted using the publicly available Dental Radiography dataset,^1^ which contains 1, 272 dental radiographs assigned to four diagnostic categories: cavities (*n* = 478), fillings (*n* = 382), implants (*n* = 252), and impacted teeth (*n* = 160). A fixed partition of 891 training images, 190 validation images, and 191 test images was used throughout. The validation subset was used for model selection and hyperparameter optimization; the test subset was reserved for final evaluation only. Each image was assigned one benchmark label, so the task was formulated as four-class image classification.

CaliDent-Net was compared with ResNet-50 (28), DenseNet-121 (32), EfficientNetV2-S (33), MobileNetV3-Large (34), ResNet-50 with SE attention (16), ResNet-50 with CBAM attention (12), ResNet-50 with SSPC, and a reimplementation of the MobileNetV2–Swin architecture described in Alsakar et al. (14). All models were trained and evaluated under matched supervised protocols, using identical data partitions, preprocessing operations, augmentation procedures, and stopping criteria. CaliDent-Net additionally incorporated label-free RACE pretraining on the training split; its contribution is therefore examined separately in the ablation study.

Implementation used Python 3.10 (Python Software Foundation Beaverton, OR, USA), PyTorch 2.1.0 [PyTorch Foundation (The Linux Foundation) San Francisco, CA USA], TorchVision 0.16.0 [PyTorch Foundation (The Linux Foundation) San Francisco, CA USA], Albumentations [Albumentations Team (open-source project, https://albumentations.ai)] (27), Optuna (Preferred Networks, Inc. Tokyo Japan) (36), SciPy [NumFOCUS, Inc. (fiscal sponsor of the SciPy project) Austin, TX USA], and scikit-learn (Inria Rocquencourt France) (35). Model training was performed on an NVIDIA RTX 3090 GPU with 24 GB of memory. Hyperparameter optimization was carried out with 60 Optuna trials using the Tree-structured Parzen Estimator sampler and median pruning. The selected configuration and the corresponding search domains are summarized in Table 2.

This benchmark is modest in scale for a deep-learning comparison study, particularly because model selection involved self-supervised pre-training and hyperparameter optimization. The reported metrics should therefore be interpreted as benchmark-specific estimates rather than as stable estimates of clinical performance. In addition, the one-label-per-image protocol excludes secondary co-occurring findings, so the results reflect dominant-label classification on a controlled benchmark rather than comprehensive radiographic reporting.

### 4.2 Implementation and hyperparameter optimization

The models were implemented in Python 3.10 using PyTorch 2.1.0, TorchVision 0.16.0, Albumentations (27), Optuna (36), SciPy, and scikit-learn (35). Training was performed on an NVIDIA RTX 3090 GPU with 24 GB of memory. Hyperparameter optimization was conducted over 60 Optuna trials using the Tree-structured Parzen Estimator sampler and median pruning. The validation subset was used as the optimization target, and the selected configuration and corresponding search domains are presented in Table 2.

CPU inference was evaluated on an Intel Core i9-12900K with CUDA disabled. Latency was measured using non-batched, single-image inference over 500 samples, preceded by 50 warm-up iterations that were excluded from the timing statistics. Peak resident memory was monitored during the same inference procedure.

### 4.3 Baseline models and training protocol

CaliDent-Net was compared with eight representative architectures: ResNet-50 (28), DenseNet-121 (32), EfficientNetV2-S (33), MobileNetV3-Large (34), ResNet-50 with SE attention (16), ResNet-50 with CBAM attention (12), ResNet-50 with SSPC, and a reimplementation of the MobileNetV2–Swin architecture described in Alsakar et al. (14). All comparison models used ImageNet initialization and were trained using identical data partitions, preprocessing operations, augmentation procedures, and supervised optimization schedules.

CaliDent-Net additionally incorporated label-free RACE pretraining on the training subset. This stage introduced further exposure to the training images and additional computational cost, although no diagnostic labels were used. Its contribution was therefore evaluated separately in the ablation study shown in Table 3. The convergence curves present only the supervised fine-tuning stage. Final results were averaged over five independent runs using matched random seeds.

**Table 3:** Ablation study evaluating the progressive performance impact of individual architectural components on CaliDent-Net.

Variant	Acc.	F1	AUC	ECE	BS
Backbone only	0.872	0.866	0.928	0.046	0.038
+SSPC	0.910	0.904	0.947	0.029	0.030
+RACE	0.932	0.928	0.962	0.025	0.027
+Ch-only (SE)	0.941	0.937	0.969	0.022	0.024
+Full PRAB	0.958	0.954	0.981	0.019	0.022
Full CaliDent-Net	0.964	0.959	0.984	**0.013**	0.021

### 4.4 Evaluation metrics

Discriminative performance was evaluated using accuracy, macro-averaged precision, macro-averaged recall, macro F1-score, and macro AUC. Predictive calibration was assessed using the Brier score, expected calibration error (ECE), maximum calibration error (MCE), and confidence sharpness, with ECE and MCE computed using 15 equal-width confidence bins. Computational efficiency was measured using GFLOPs and CPU latency. CPU latency was evaluated under a non-batched single-image inference protocol and reported descriptively.

### 4.5 Statistical analysis

Statistical analyses were performed using run-level results from five independent training runs with matched random seeds. Performance results in Tables 3, 4 are represented as mean ± standard deviation (SD). For comparisons between CaliDent-Net and each baseline model, seed-wise accuracy values were analysed using two-sided paired t-tests. The paired design was selected because all models were trained and evaluated using the same data partitions and matched random seeds, thereby accounting for seed-related variability. Holm-Bonferroni correction was applied across multiple baseline comparisons to control the family-wise error rate at α = 0.05.

**Table 4:** Discriminative performance on the Dental Radiography test set, reported as mean ± SD over five runs.

Model	Accuracy	Precision	Recall	F1	AUC
ResNet-50^*^	0.869 ± 0.015	0.871 ± 0.019	0.858 ± 0.022	0.864 ± 0.020	0.916 ± 0.013
DenseNet-121^*^	0.884 ± 0.012	0.887 ± 0.015	0.876 ± 0.018	0.881 ± 0.016	0.929 ± 0.011
EfficientNetV2-S^*^	0.908 ± 0.010	0.911 ± 0.013	0.899 ± 0.015	0.905 ± 0.013	0.944 ± 0.010
MobileNetV3-Large^*^	0.878 ± 0.014	0.875 ± 0.017	0.866 ± 0.019	0.870 ± 0.018	0.912 ± 0.014
ResNet-50 + SE^*^	0.916 ± 0.009	0.918 ± 0.012	0.908 ± 0.014	0.913 ± 0.012	0.953 ± 0.009
ResNet-50 + CBAM^*^	0.923 ± 0.008	0.925 ± 0.011	0.916 ± 0.013	0.920 ± 0.011	0.958 ± 0.008
ResNet-50 + SSPC^*^	0.934 ± 0.007	0.937 ± 0.010	0.929 ± 0.012	0.933 ± 0.010	0.967 ± 0.007
MobileNetV2 + Swin (14)^*^	0.941 ± 0.006	0.943 ± 0.009	0.935 ± 0.011	0.939 ± 0.009	0.971 ± 0.006
CaliDent-Net	0.964 ± 0.004	0.961 ± 0.006	0.957 ± 0.007	0.959 ± 0.006	0.984 ± 0.003

^*^ denotes significance after Holm-Bonferroni correction (α = 0.05). The proposed model (CaliDent-Net) obtained the best result for each metric. BS, Brier score; ECE, expected calibration error; MCE, maximum calibration error; Sharp., confidence sharpness.

For the ablation study in Table 5, the six model variants were compared using one-way analysis of variance (ANOVA), followed by Tukey's honestly significant difference (HSD) test for *post-hoc* pairwise comparisons. Effect sizes were reported using Cohen's d for paired model comparisons and η^2^ for the ablation analysis. Because the number of independent runs was limited (*n* = 5), statistical significance was interpreted together with absolute performance differences, SDs, and effect sizes.

**Table 5:** Test set calibration metrics (mean±SD) across five independent runs for the evaluated models.

Model	BS	ECE	MCE	Sharp.
ResNet-50	0.038 ± 0.004	0.046 ± 0.005	0.093 ± 0.009	0.47
DenseNet-121	0.035 ± 0.003	0.040 ± 0.005	0.084 ± 0.008	0.49
EfficientNetV2-S	0.033 ± 0.003	0.037 ± 0.004	0.076 ± 0.007	0.51
MobileNetV3-Large	0.040 ± 0.004	0.048 ± 0.006	0.097 ± 0.010	0.45
ResNet-50 + SE	0.029 ± 0.003	0.032 ± 0.004	0.068 ± 0.007	0.52
ResNet-50 + CBAM	0.027 ± 0.003	0.029 ± 0.004	0.061 ± 0.006	0.53
ResNet-50 + SSPC	0.024 ± 0.002	0.022 ± 0.003	0.052 ± 0.006	0.54
MobileNetV2 + Swin (14)	0.022 ± 0.002	0.019 ± 0.003	0.047 ± 0.005	0.55
CaliDent-Net	0.021 ± 0.002	0.013 ± 0.004	0.039 ± 0.010	0.56

Full CaliDent-Net denotes the complete proposed configuration. Acc., accuracy; F1, macro-averaged F1-score; AUC, macro-averaged area under the receiver operating characteristic curve; ECE, expected calibration error; BS, Brier score.

## 5 Results

This section presents the classification and calibration performance of CaliDent-Net, f.ollowed by the ablation and label-efficiency analyses. The learned representations, qualitative explanations, and computational requirements are subsequently examined to provide a broader assessment of model behavior.

### 5.1 Classification performance

**Figure 2:**
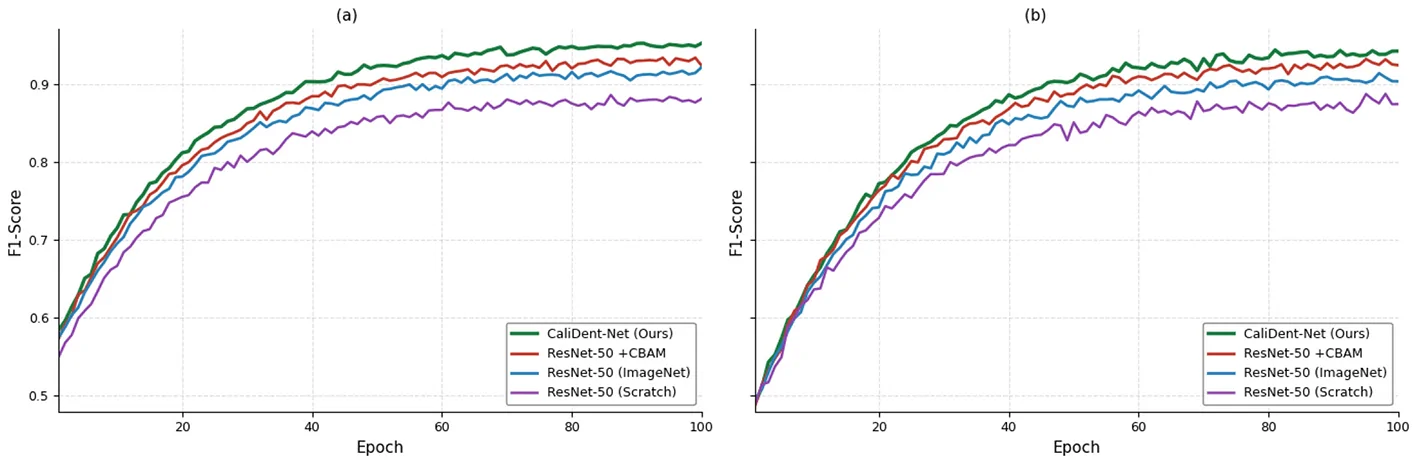
Evolution of training and validation macro F1-scores across epochs. **(a)** Training, **(b)** Validation.

**Figure 3:**
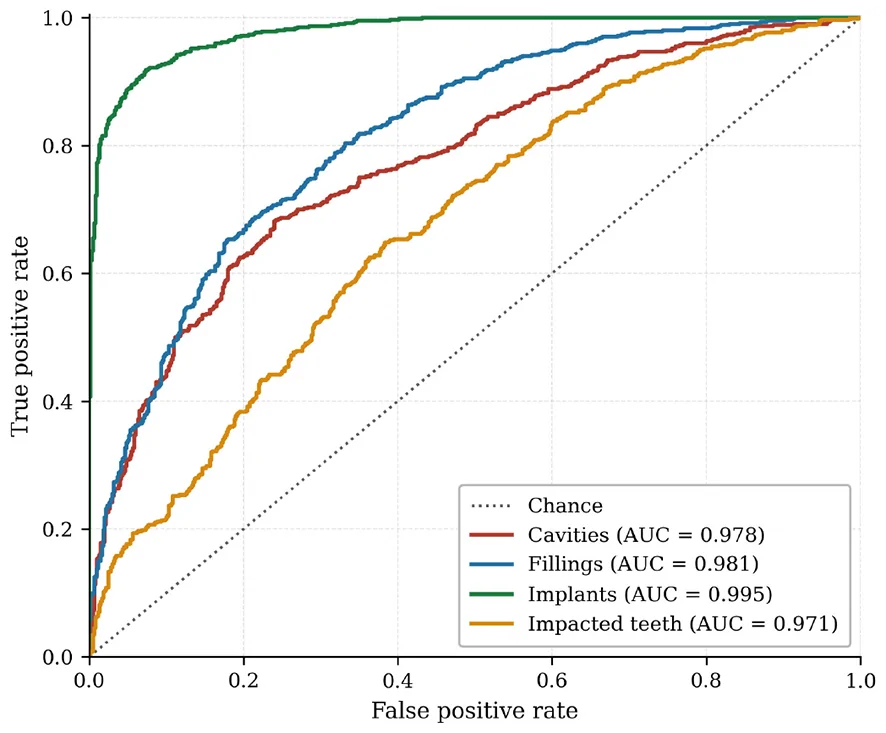
Multiclass ROC curves demonstrating robust classification performance across all evaluated dental pathologies.

Table 3 shows the classification performance across five independent runs. CaliDent-Net achieved the highest accuracy (0.964 ± 0.004), macro F1-score (0.959 ± 0.006), and macro AUC (0.984 ± 0.003). The strongest comparison model, MobileNetV2 + Swin, obtained an accuracy of 0.941 ± 0.006, yielding an absolute difference of 2.3 percentage points. On the 191-image test set, this difference corresponds to approximately four additional correctly classified radiographs. All comparisons with CaliDent-Net remained statistically significant after Holm-Bonferroni correction at the family-wise significance level of α = 0.05. The training and validation trajectories in Figure 2 followed similar patterns throughout the 100 supervised epochs, with no marked divergence during the later stages of optimization. CaliDent-Net also maintained a higher macro F1-score than the ResNet-50 variants after convergence. The one-versus-rest ROC curves in Figure 3 show class-specific AUC values ranging from 0.971 for impacted teeth to 0.995 for implants, indicating consistently strong discrimination across the four diagnostic categories.

### 5.2 Calibration performance

Calibration results are represented in Table 4. CaliDent-Net obtained the lowest Brier score (0.021 ± 0.002), ECE (0.013 ± 0.004), and MCE (0.039 ± 0.010) among the evaluated models. Relative to ResNet-50, these values correspond to reductions of approximately 45% in Brier score and 72% in ECE. An ECE of 0.013 indicates an average absolute difference of approximately 1.3 percentage points between confidence and empirical accuracy across the predefined confidence bins. This quantity represents an average calibration discrepancy and should not be interpreted as a guarantee for every confidence interval or individual prediction.

**Figure 4:**
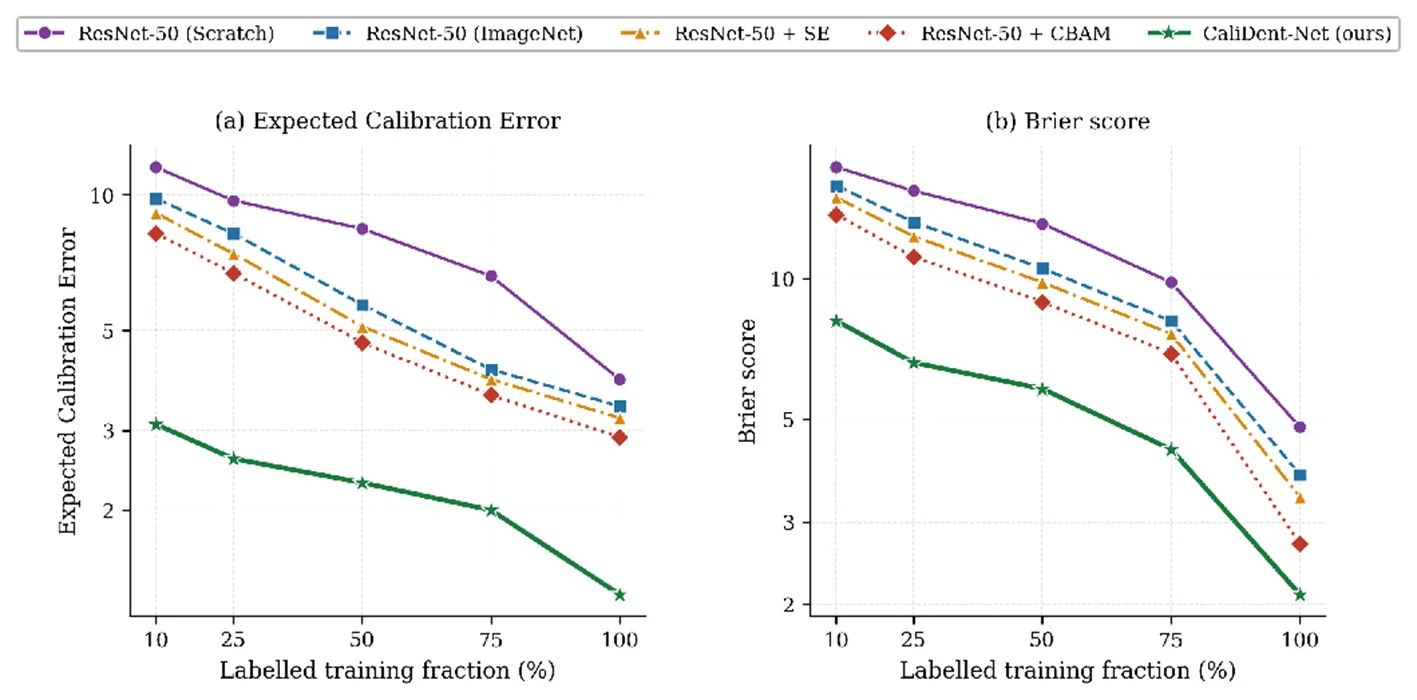
Comparison of **(a)** expected calibration error and **(b)** Brier score across different fractions of labeled training data for the evaluated models.

**Figure 5:**
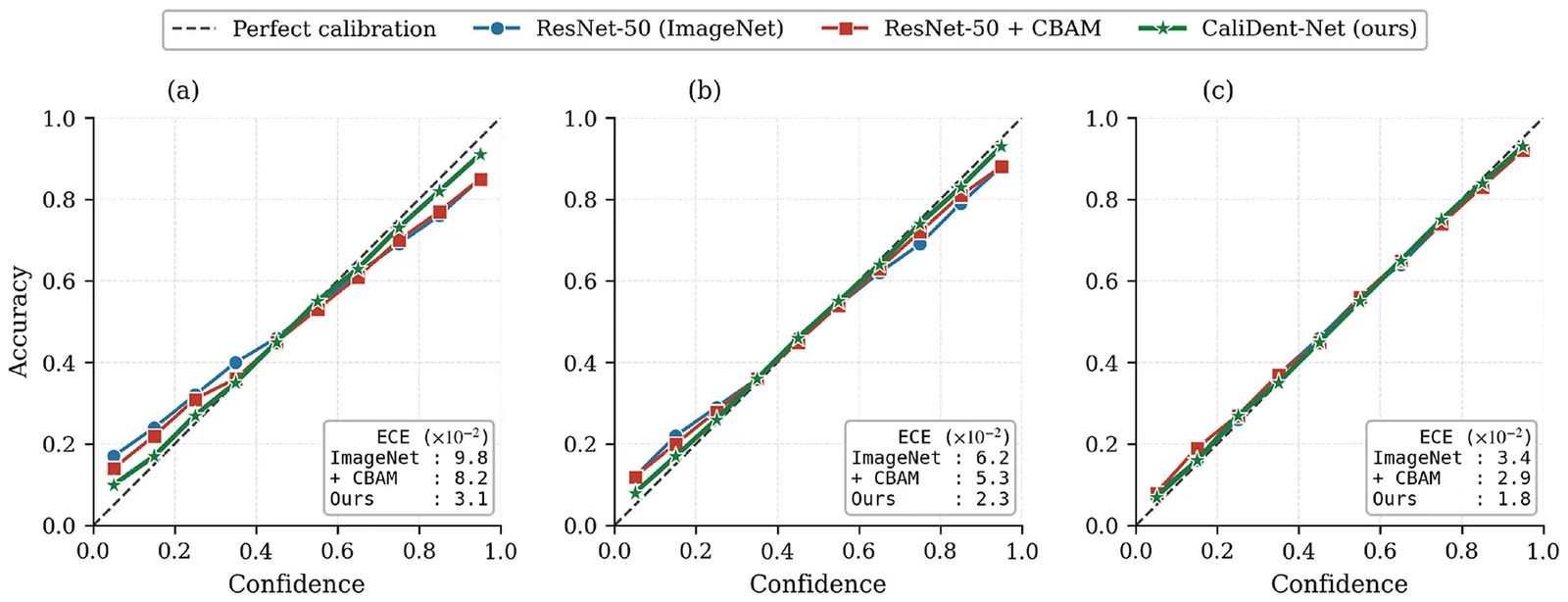
Reliability diagrams and ECE values for ResNet-50 (ImageNet), ResNet-50 + CBAM, and CaliDent-Net across varying training data fractions. **(a)** 10% labeled data. **(b)** 50% labeled data. **(c)** 100% labeled data.

Figure 4 examines calibration across reduced labeled-data conditions. CaliDent-Net produced the lowest ECE and Brier score at each evaluated fraction. The reliability diagrams in Figure 5 provide a complementary comparison at 10%, 50%, and 100% labeled data. Across these settings, the CaliDent-Net curve remained closer to the perfect-calibration diagonal than the two ResNet-50 comparison models.

### 5.3 Ablation analysis

Table 5 evaluates the incremental contribution of the proposed components. Introducing SSPC increased accuracy from 0.872 to 0.910 and reduced ECE from 0.046 to 0.029. RACE pre-training further increased accuracy to 0.932, while channel-only attention produced an accuracy of 0.941. Replacing the single channel-attention stream with the complete PRAB configuration increased accuracy to 0.958 and reduced ECE to 0.019. The complete CaliDent-Net configuration achieved the highest reported discrimination and the lowest calibration errors.

The global ablation comparison was significant, with *F*(5, 24) = 119.7 and *p* < 0.001. The comparison between the full PRAB and channel-only attention variants also remained significant after Tukey's adjustment, with *q*(5, 24) = 6.32 and *p* < 0.001. These results support the incremental contribution of the proposed components under the present experimental protocol.

### 5.4 Label efficiency

Table 6 reports classification accuracy as the proportion of labeled training data was reduced. CaliDent-Net achieved the highest accuracy at every evaluated fraction. With 20% of the available labels, it reached an accuracy of 0.879, exceeding ResNet-50, EfficientNetV2-S, and MobileNetV2 + Swin by 11.1, 7.2, and 5.5 percentage points, respectively. When trained with 40% of the labels, CaliDent-Net achieved an accuracy of 0.928, which exceeded the 0.869 obtained by ResNet-50 using the complete labeled training set. The corresponding seed-matched comparison yielded *t*(4) = 6.9 and *p* < 0.005.

**Table 6:** Classification accuracy across labeled training fractions, reported as mean ± SD over five independent runs.

Model	10%	20%	40%	75%	100%
ResNet-50	0.712 ± 0.022	0.768 ± 0.018	0.826 ± 0.015	0.852 ± 0.013	0.869 ± 0.015
EfficientNetV2-S	0.758 ± 0.019	0.807 ± 0.016	0.856 ± 0.014	0.889 ± 0.011	0.908 ± 0.010
ResNet-50 + CBAM	0.781 ± 0.017	0.824 ± 0.014	0.876 ± 0.012	0.907 ± 0.010	0.923 ± 0.008
MobileNetV2 + Swin (14)	0.784 ± 0.016	0.824 ± 0.013	0.892 ± 0.011	0.925 ± 0.009	0.941 ± 0.006
CaliDent-Net	0.841 ± 0.012	0.879 ± 0.010	0.928 ± 0.008	0.952 ± 0.006	0.964 ± 0.004

### 5.5 Interpretability analysis

**Figure 6:**
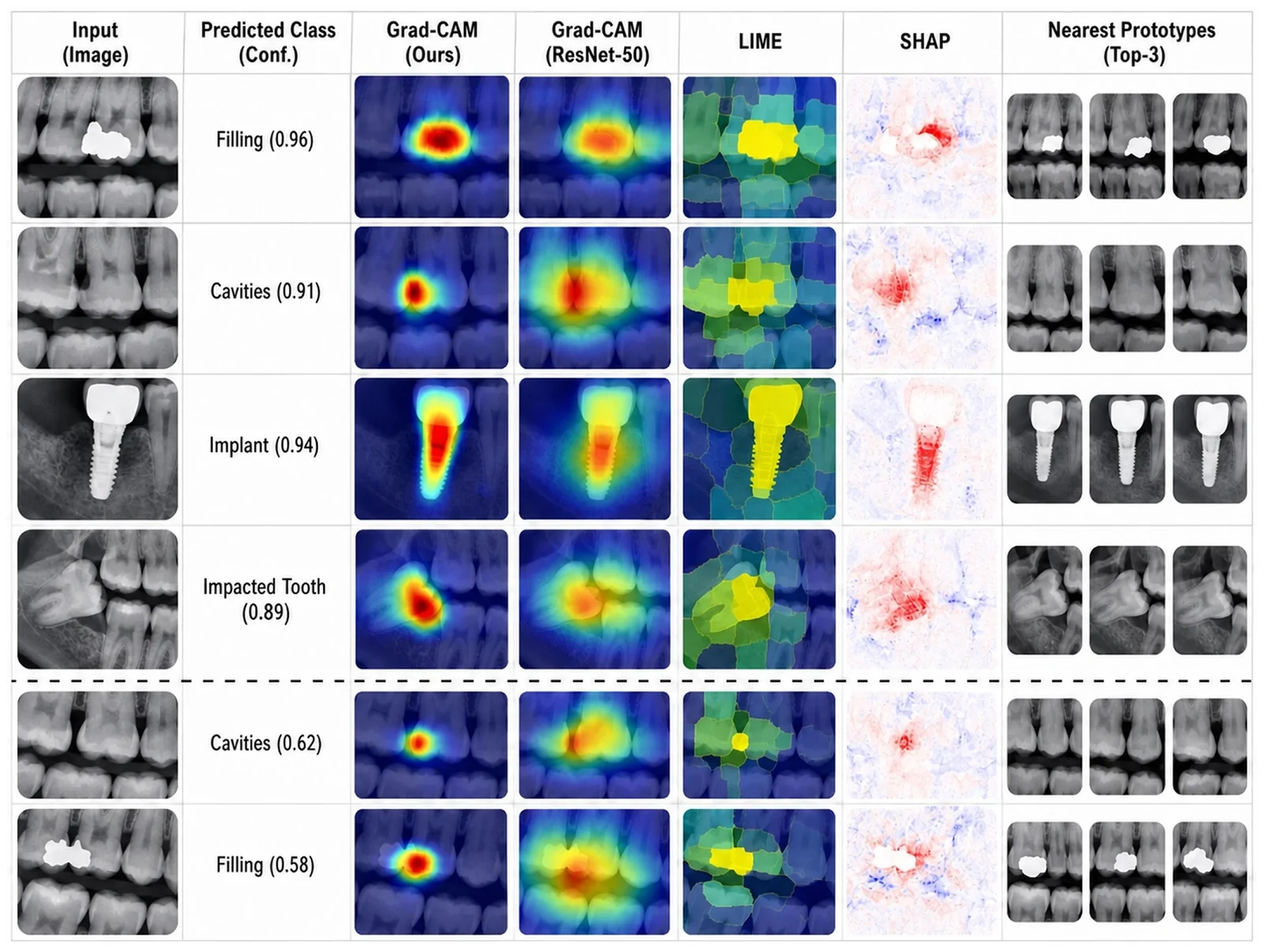
Qualitative comparison of visual explanation methods, attribution maps, and top-3 retrieved prototypes for representative dental radiographs.

Interpretability was examined using case-based prototype retrieval and pixel-attribution methods. Figure 6 presents six representative test cases, including one high-confidence example from each class and two lower-confidence cases involving cavities and fillings. For each radiograph, the figure reports the predicted class and confidence, Grad-CAM maps generated by CaliDent-Net and ResNet-50, LIME and SHAP explanations, and the three nearest prototypes retrieved from the training set. Prototype retrieval identifies similar reference cases, whereas the attribution methods indicate the image regions that contributed most strongly to the prediction.

A preliminary face-validity review was performed on 40 randomly selected prototype retrievals. One non-blinded clinical co-author judged 38 retrievals to be consistent with the predicted diagnostic category; the two disagreements involved low-contrast cavity and filling cases. This assessment should be interpreted cautiously because it involved one non-independent rater, a small sample, and no blinded evaluation or inter-rater agreement analysis.

**Figure 7:**
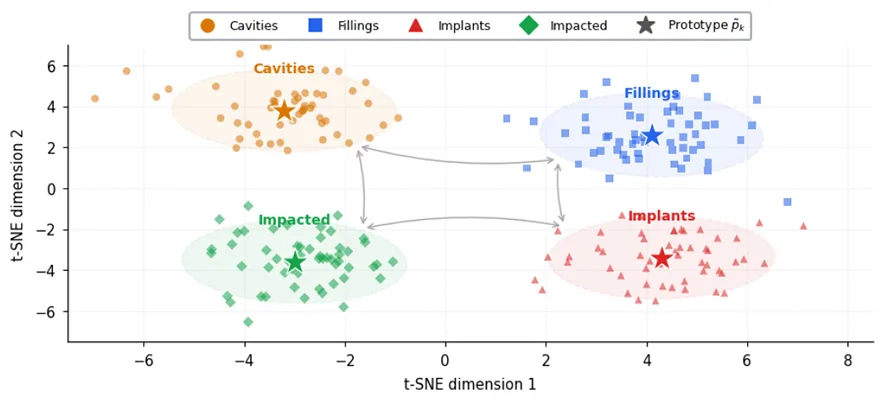
Two-dimensional t-SNE visualization of the SSPC embedding space. Stars indicate the learned class prototypes, and shaded ellipses represent class-wise covariance regions.

The t-SNE projection in Figure 7 provides a qualitative view of the SSPC embedding space. The diagnostic categories form broadly distinct clusters, and the corresponding prototypes are positioned near the central regions of their class distributions. Because t-SNE may distort global distances, the projection is treated as a visual consistency check rather than quantitative evidence of class separability.

The interpretability evidence remains preliminary. The benchmark provides image-level labels rather than lesion-level annotations, so localization accuracy cannot be quantified. Stronger evidence would require independent blinded dental readers, a predefined explanation-quality rubric, inter-rater agreement statistics, and lesion-level reference annotations suitable for quantitative localization assessment.

### 5.6 Computational efficiency

The computational profiles of the evaluated models are reported in Table 7. CaliDent-Net required 2.8 GFLOPs. Mean CPU inference latency was 214 ms per image under the non-batched evaluation protocol. Although slower than MobileNetV3-Large and the conventional ResNet-50 backbone, this latency supports retrospective, non-real-time analysis on the evaluated workstation. These measurements describe computational feasibility on the specified hardware and do not establish performance within a deployed clinical system.

**Table 7:** Comparison of computational complexity and CPU latency across the evaluated models.

Model	GFLOPs	CPU latency (ms)
ResNet-50	4.1	182
DenseNet-121	5.7	241
EfficientNetV2-S	2.8	178
MobileNetV3-Large	0.2	68
ResNet-50 + SE	4.1	195
ResNet-50 + CBAM	4.2	204
MobileNetV2 + Swin (14)	4.2	208
CaliDent-Net	2.8	214

CaliDent-Net is the model proposed in this study. Its values identify the proposed model rather than the best result obtained for each metric. GFLOPs, billions of floating-point operations per forward pass; CPU, central processing unit.

## 6 Discussion

The ablation findings indicate that CaliDent-Net benefits from the combined effects of domain-specific self-supervised pretraining, prototype-based classification, attention refinement, and temperature scaling. SSPC produced the largest initial improvement over the backbone, while RACE and PRAB yielded further gains in discriminative performance and calibration. Temperature scaling subsequently reduced ECE from 0.019 to 0.013, indicating that most of the calibration improvement was achieved by the learned model before *post-hoc* adjustment. CaliDent-Net also surpassed the full-label performance of ResNet-50 when fine-tuned with only 40% of the labeled data, suggesting improved label efficiency under the present benchmark protocol. In addition, the measured CPU latency of 214 ms per image supports retrospective, non-real-time inference on the evaluated workstation.

### 6.1 Statistical and practical interpretation

The seed-matched statistical comparisons indicate that CaliDent-Net achieved consistent accuracy improvements over the evaluated baselines after Holm–Bonferroni correction. However, because the inferential analysis was based on five independent runs and a 191-image test set, these findings should be interpreted as benchmark-level evidence rather than evidence of clinical benefit. The 2.3 percentage-point improvement over the strongest baseline corresponds to approximately four additional correctly classified radiographs. Therefore, model performance was interpreted using statistical significance together with absolute differences, standard deviations, effect sizes, calibration metrics, and computational requirements rather than *p*-values alone. Whether improvements of this magnitude affect diagnostic decisions, referral patterns, or patient outcomes requires adequately powered prospective clinical studies with predefined clinical endpoints.

### 6.2 Dataset scope and generalizability

The study was conducted on a single public dataset containing 1, 272 radiographs, with 191 images reserved for testing. This sample size is modest relative to the complexity of the proposed framework and the extent of model optimization. Self-supervised pretraining was restricted to the training partition, hyperparameters were selected using the validation set, and the results were averaged across five independent runs. The close training and validation trajectories in Figure 2 provide no clear evidence of late-stage overfitting. Nevertheless, these precautions cannot exclude dataset-specific optimization. Differences in scanner vendors, exposure parameters, acquisition procedures, image quality, disease prevalence, and patient characteristics may alter performance substantially (23). Independent multi-center validation is therefore required before claims of broader robustness or clinical generalizability can be made.

### 6.3 Single-label task formulation

The benchmark assigns one dominant class to each radiograph, although multiple dental findings may coexist in routine practice. Under this formulation, secondary abnormalities are neither learned nor included in the evaluation. The reported performance therefore reflects dominant-label classification rather than comprehensive radiographic interpretation. A clinically faithful extension should adopt native multi-label prediction, for example, through independent class probabilities, per-label calibration, and multiple prototypes capable of representing concurrent findings.

### 6.4 Interpretability

The prototype retrieval, Grad-CAM, LIME, and SHAP examples provide complementary qualitative views of model behavior. Prototype retrieval identifies similar training cases, whereas attribution methods indicate image regions that influenced the prediction. However, the current evidence does not establish clinical interpretability or explanation fidelity. The prototype review involved a small, non-blinded assessment by one co-author, and lesion-level annotations were unavailable for quantitative localization analysis. Future evaluation should include independent blinded dental readers, a predefined scoring protocol, inter-rater agreement, and lesion-level reference annotations for quantitative comparison of prototype retrieval and attribution methods (24–26).

### 6.5 Limitations and future directions

The principal limitations are the single-dataset design, modest cohort size, single-label formulation, and qualitative interpretability assessment. In addition, the bounded similarity scores produced by SSPC have not been evaluated formally for out-of-distribution detection, and a single prototype per class may not capture the full visual diversity within each diagnostic category. Future work should therefore prioritize external multi-institutional validation, native multi-label learning, multi-prototype class representations, explicit out-of-distribution assessment, blinded reader studies, and privacy-preserving collaborative training. Integration with dental charting or decision-support systems should be considered only after these validation requirements have been addressed (37).

Overall, CaliDent-Net provides evidence of improved discrimination, calibration, and data efficiency on a controlled dental-radiography benchmark. These findings support the technical feasibility of the proposed framework, but they do not establish clinical readiness, cross-institutional robustness, or superiority in prospective dental practice.

## 7 Conclusion

This study introduced CaliDent-Net, a unified framework that brings together domain-constrained self-supervised pretraining (RACE), forward-independent dual-stream recalibration attention (PRAB), and cosine-prototype calibrated classification (SSPC) to address data scarcity, miscalibration, decision opacity, and computational cost in dental radiograph classification within a single pipeline. On the Dental Radiography benchmark, it reaches 96.4% accuracy and an ECE of 0.013, outperforms eight competitive baselines, and matches the best baseline's full-data accuracy using approximately 40% of the labels. The most useful finding is that a meaningful share of the calibration improvement is structural: Bounding the logit space through cosine normalization cuts ECE relative to ResNet-50 before any *post-hoc* correction is applied. These results come from a single benchmark of 1, 272 images and from a single-rater, non-blinded interpretability check, so they should be read as evidence of technical viability rather than of clinical readiness. External multi-site validation, a multi-label reformulation, and a blinded multi-rater interpretability study are prerequisites before the approach could be considered for evaluation as a second-reader decision-support aid. With those caveats, the combination of architectural calibration and CPU-level inference makes CaliDent-Net a practical starting point for that future work.

## Data Availability

Publicly available datasets were analyzed in this study. This data can be found here: https://www.kaggle.com/datasets/Dental Radiography.

## Footnotes

1
https://www.kaggle.com/datasets/imtkaggleteam/dental-radiography
